# Uptake of nanowires by human lung adenocarcinoma cells

**DOI:** 10.1371/journal.pone.0218122

**Published:** 2019-06-21

**Authors:** Laura Abariute, Mercy Lard, Elke Hebisch, Christelle N. Prinz

**Affiliations:** 1 Division of Solid State Physics, Lund University, Lund, Sweden; 2 NanoLund, Lund University, Lund, Sweden; LAAS-CNRS, FRANCE

## Abstract

Semiconductor nanowires are increasingly used in optoelectronic devices. However, their effects on human health have not been assessed fully. Here, we investigate the effects of gallium phosphide nanowires on human lung adenocarcinoma cells. Four different geometries of nanowires were suspended in the cell culture for 48 hours. We show that cells internalize the nanowires and that the nanowires have no effect on cell proliferation rate, motility, viability and intracellular ROS levels. By blocking specific internalization pathways, we demonstrate that the nanowire uptake is the result of a combination of processes, requiring dynamin and actin polymerization, which suggests an internalization through macropinocytosis and phagocytosis.

## Introduction

The use of nanoscaled components in semiconductor technology enabled a substantial improvement in electronic device performance[1]. For instance, III-V semiconductor nanowires are high aspect ratio nanostructures that have been studied extensively and that are considered a promising material for developing optoelectronic devices [2]. Better efficiency light emitting diodes and solar cells have been produced using III-V nanowires [3,4]. The advantages of using nanowires come from the possibility to fabricate highly controlled single crystalline materials with tunable geometry and crystalline structure [5–7]. There is a growing concern about possible nanowire exposure and its impact on human health and the environment. The main focus of concern being nanowire geometry, which resembles that of asbestos fibers and carbon nanotubes. Most of the current research has been concentrated on nanowire arrays and their interactions with living cells [8–13], as well as their applications in biosensing and drug delivery [14–20]. There are only a handful of studies on the effects of substrate-free semiconductor nanowires on biological tissue and ecosystems. *In vitro* exposure of rat alveolar macrophages to silicon (SiNW) nanowires showed no significant increase in reactive oxygen species levels [21]. *In vivo* exposure to SiNW via instillation in rats showed a transient dose-dependent increase of lung injury and inflammation[22]. In two studies of gallium phosphide (GaP) and gallium indium phosphide (GaInP) nanowires [23,24], we have found that nanowire exposure through ingestion do not have any detrimental effects on viability and tissue function in *Daphnia magna* and *Drosophila melanogaster* [25,26]. Although, few studies present any adverse effects of semiconductor nanowires, we have shown that 5 μm and 10 μm nanowires injected in the rat brain induce a sustained tissue inflammation even one year after the injection [27,28]. Therefore, more studies are necessary to complete the knowledge on the safety of semiconductor nanowires. To date, there are no available commercial products containing nanowires since their synthesis suffers from low throughput and is expensive [5]. However, new technologies, such as aerotaxy, will enable large scale production of III-V nanowires, such as GaP nanowires, in the near future [29]. Integrated in an optoelectronic device such as a solar cell or a LED, these III-V nanowires would be coated with a thin oxide layer for passivation [30]. Therefore, there is a need for investigating the effects of oxide-coated III-V nanowires on cells, and their cellular internalization.

In this paper, we investigate the effects of aluminum oxide-coated GaP nanowires on A549 human lung adenocarcinoma cells in culture. These cells, although cancerous, have been used in numerous nanosafety studies [31–34] and provide a first step towards understanding the effects of nanowires on lung cells. Moreover, these cells have been used as a standardized model to study drug metabolism in lungs [35]. We exposed cells to different geometries of free-floating nanowires in solution, and assessed the nanowire distribution in cells as a function of time, as well as the nanowire effects on cell proliferation. Moreover, for a specific nanowire geometry, we have assessed the cell viability and production of reactive oxygen species (ROS). The nanowire uptake mechanism was investigated by inhibiting different internalization pathways. Our results show that nanowires suspended in the cell medium are not acutely toxic to human lung adenocarcinoma cells. When suspended in the cell medium, nanowires are taken up through phagocytosis and macropinocytosis. However, we could not find any nanowires in early endosomes or lysosomes.

## Materials and methods

### GaP nanowire fabrication

GaP nanowires were synthesized using gold-particle assisted metal organic vapor phase epitaxy (MOVPE). Au seed nanoparticles of 60 nm and 80 nm in diameter were deposited randomly on a GaP (111)B substrate by aerosol technology[36], at a density of 1 particle/μm^2^. The samples were placed in a MOVPE reactor (Aixtron 200/4), and heated to 650°C for 10 min in H_2_/PH_3_ (AGA AB) atmosphere to remove the native oxide, anneal the Au particles to the GaP substrate and form a liquid-solid crystallization interface. The temperature was subsequently reduced to 440°C and the nanowire growth was initiated by introducing the gas precursor trimethylgallium (TMGa, SAFC Hitech) in the growth chamber. Undesirable radial growth was prevented by introducing HCl (AGA AB) into the growth chamber throughout the whole growth time [37]. Nanowires of 1 and 5 μm in length were fabricated in this study.

### GaP nanowire surface treatment and characterization

To obtain a homogeneous surface chemistry, we have coated the nanowires with a thin layer of Al_2_O_3_.The substrates with nanowires were placed in a Savanah-100 ALD reactor, which was heated to 250°C and alternating pulses of trimethylaluminium (Strem chemicals Inc.) and deionized H_2_O (Milllipore) vapor were introduced. The cycles were repeated until 5 nm of Al_2_O_3_ were deposited. We have subsequently determined NWs diameter using SEM for imaging and the Image J manual measurement tool for analysis. Additionally, we have confirmed Al_2_O_3_ presence on the surface using Raman spectroscopy (S1 Fig). Raman spectrum was excited using 725 nm laser with power set at 2.3 mW and acquired for 20 s at room temperature. The analysis was performed by fitting the spectrum with Lorentzian functions using the software Origin.

### Cell line

Human lung adenocarcinoma A549 cells (at passage #91) were purchased from European Collection of Cell Cultures (ECCC, distributor Sigma Aldrich). A549 cells were seeded at a density of 7000 cells/cm^2^ in T-75 culture flasks. They were cultured in Ham’s F12K media (Gibco), supplemented with 10% fetal bovine serum (FBS, Sigma Aldrich) and 1% Penicillin-Streptomycin (BioReagent, Sigma Aldrich).

Prior to the nanowire exposure, cells were seeded at 1.2 x 10^4^ cells/cm^2^ in a 35 mm Petri dish with a collagen-coated glass coverslip at the bottom. Cells were let to adhere and adapt to the environment for 24 h.

Collagen-coated glass coverslips were prepared as follows: coverslips were sterilized under UV light for 10 min and incubated in 100 mg/mL collagen water solution (Corning) for 24 hours at room temperature in a sterile laminar flow hood. Collagen coated-coverslips were washed once in Ham’s F-12K media, and placed on the bottom of the 35 mm Petri dish.

### Exposure to nanowires and characterization

Nanowire substrates were sterilized in 70% EtOH for 10 min and left to dry in a sterile laminar flow hood for 24 h. Nanowires were suspended in sterile deionized water (18.2 MΩ.cm, Millipore) at a concentration of 1.38 × 10^−8^ m^2^/μL using sonication. This concentration was achieved by calculating the total area of the nanowires on each substrate (by measuring the density and dimensions of the nanowires on each substrate) and adjusting correspondingly the amount of water the nanowires are suspended in, in order to reach the desired concentration. The length and diameter of the suspended nanowires were characterized after sonication using SEM (LEO 1560, Zeiss, Germany) by depositing a 0.2 μL drop of nanowire suspension on a silicon wafer, drying it and imaging the nanowires. At least 200 nanowire were measured for each samples using ImageJ [38]. A volume of 150 μL of the nanowire suspension was added to 2.85 mL of cell culture. (corresponding to a total nanowire surface area of 2.07 × 10^−6^ m^2^). After 48 h, the cells were fixed and stained as described below.

### Immunofluorescence

Cells were fixed using 2% paraformaldehyde (PFA) for 10 min, 4% PFA for 20 min, and washed 3 times in PBS for 10 min. Cells were permeabilized and unspecific binding was blocked by incubating them in PBS with 0.25% Triton X-100 and 2% bovine serum albumin (BSA, Sigma Aldrich) for 10 min, followed by 3 washes in PBS for 10 min. The cells were subsequently incubated in a 4 μg/mL goat anti- LAMP-1 (Thermo Fisher) and 1% BSA solution in PBS for 90 min and washed 3 times in PBS for 10 min. The samples were then incubated in a 4 μg/mL anti-goat AlexaFluor 594 (Thermo Fisher) and 1% BSA solution in PBS for 60 min in the dark at room temperature, and subsequently washed 3 times in PBS for 10 min. The cell actin was stained by incubating cells in a 0.033 μM AlexaFluor 488 Phalloidin (Thermo Fisher) and 1% BSA PBS solution, for 90 min in the dark, at room temperature, and washed 3 times in PBS for 10 min. Cells incubated in 1μg/mL Hoechst 33342 (Sigma Aldrich) in PBS for 2 min and washed 3 times in PBS for 10 min, for counterstaining.

Cover slips were mounted on a clean microscope slide using MOWIOL 4–88 solution (Merck), containing 2% DABCO anti-fading agent (Sigma Aldrich). Mounted samples were left at room temperature in the dark overnight for MOWIOL 4–88 to cure and stored at +4°C for short term storage (up to one week), or– 20°C for longer storage.

### Confocal microscopy

Confocal lateral (“XY”) and axial (“XZ”) scans of fixed A549 cells fluorescence labelled for cytoskeletal F-actin using the compound Phalloidin-STAR635P (Abberior GmbH, Goettingen, Germany), for the nucleus using Hoechst33342 (Sigma-Aldrich via Merck KGaA, Darmstad, Germany) and incubated with GaP nanowires were acquired using an Abberior 2C STED 775 QUAD Scan microscope (Abberior Instruments GmbH, Goettingen, Germany) operated in confocal mode (i.e. without switching on the STED laser). The Phalloidin-STAR645P signal was excited with a laser line at 640 nm and detected in a spectral window of (650–720) nm; the Hoechst33342 nuclear signal was excited with a laser line at 405 nm and detected in a spectral window of (425–475) nm. Both fluorescence colour channel signals were recorded using Avalanche Photo Diodes (APD) equipped with the appropriate filter sets. The nanowire signal was detected by a PhotoMultiplier Tube (PMT) onto which the reflected 640 nm illumination light was directed via a pellicle beam splitter in the beampath of the microscope. The pixel size was (50 x 50) nm2 for the XY-scans and (50 x 250) nm2 for the XZ-scans. The pinhole was set to 1.7 Airy units and the samples were scanned with a pixel dwell time of 10 μs.

### Cell viability and ROS assays

The cell viability was assessed using fluorescein diacetate (FDA, Sigma Aldrich) and propidium iodide (PI, Sigma Aldrich) according to a protocol acquired from *Ibidi* GmbH[39]. The cell medium was removed and replaced by fresh FDA (8 μg/mL) and PI (20 μg/mL) solution, in Ham’s F12K cell medium without fetal bovine serum (FBS) and phenolphthalein, for 5 min in the dark at room temperature. Afterwards, cells were washed once in warm PBS, and imaged immediately in Ham’s F12K media without FBS and phenolphthalein, using fluorescence microscopy (Nikon Eclipse T*i* microscope). Ten images (of area 1.78 mm^2^ each) were counted. Positive controls were fixed in 4% PFA for 30 min.

ROS levels in cells were assessed using the Image-iT LIVE Green Reactive Oxygen Species detection kit assay (Thermo Fisher) according to the protocol provided by the manufacturer[40]. In short, cells were washed in warm Hank’s Buffer Salt Solution (HBSS) without indicator and incubated in a 25 μM carboxy-H_2_DCFDA solution at 37°C for 30 min, in the dark. During the last 5 min of incubation, the cell DNA was counterstained with Hoeschst 33342, as described in the *immunofluorescence* section above. Cells were then carefully washed in warm HBSS and imaged immediately using fluorescence microscopy (Nikon Eclipse T*i* microscope). Ten images (of area 1.78 mm^2^ each) were counted. Positive ROS controls were prepared by incubating cells with 100 μM *tert*-butyl hydroperoxide in fully supplemented Ham’s F12K media for 24 hours and washed twice with warm HBSS prior to performing the ROS assay.

### Nanowire quantification

We have chosen to use optical microscopy, as opposed to transmission tunneling microscopy (TEM) to visualize nanowires in cells for two reasons: (i) TEM requires sectioning, which is not well suited to tissue containing GaP nanowires since the nanowires are brittle and (ii) using optical microscopy allowed us to assess a great number of cells (hundreds) as opposed to TEM. The nanowire uptake was determined using brightfield microscopy (Nikon Eclipse T*i* microscope) and n = 17 images (x100 magnification), were evaluated and more than 100 cells in each group were analyzed. The total count of cells, cells with engulfed nanowires, nuclei count, nuclei shape, nanowire count, nanowire dispersion and location were determined by using the open source software ImageJ [38] and the Cell Count plugin. In some cases, brightfield images were inverted using ImageJ, in order to make nanowires more visible when overlaid with fluorescence microscopy images (e.g. see S4 Fig).

### Phase holographic imaging

The cell motility and migration were tracked using a HoloMonitor M4 microscope (Phase Holographic Imaging AB, Sweden). Petri dishes with A549 samples were placed on a HoloMonitor and were monitored until 48 h after the beginning of the nanowire exposure. Images were saved every 5 min. All experiments were repeated 3 times. The cell motility (full travelled distance for a cell from the time = 0 s to the end time point) and migration (shortest travelled distance during this time) were determined using the HoloStudio software, according to the manual. In each set of time-lapse images, 10 cells were tracked for 48 h from the beginning of the nanowire exposure (n = 30 for both exposure and control).

### Determination of the nanowire uptake mechanism

To evaluate the nanowire uptake dynamics, A549 cells were seeded on a sterile collagen-coated glass cover slip and let to adjust for 24 hours. Cells were then exposed to 80 nm diameter, 5 μm length nanowires for 1, 4, 8, 24, and 48 hours. Cells were subsequently fixed, stained for cell F-actin and DNA, and imaged using fluorescent microscopy. Nanowires were imaged using brightfield microscopy as described above.

Selected uptake mechanisms were blocked by adding cytochalasin D (Cayman Chemicals, USA; final concentration 0.005 μM), dynasore (Cayman Chemicals, USA; final concentration 100 μM), chlorpromazine (Cayman Chemicals, USA; final concentration 2.5 μM), or nystatin (Alfa Aesar, Germany; final concentration 25 μM) into the cell medium after 4 hours of exposure to nanowires. Stock solutions of cytochalasin D, dynasore, chlorpromazine and nystatin were made in DMSO. The final DMSO concentration in the A549 cell culturing media was below 0.02%. The concentration were chosen after testing a range of four different concentrations found in the literature [41–45]. For each compound, we chose the highest concentration for which the cell morphology was not affected. When adding the blocking chemicals, special care was taken in avoiding stirring the medium in order to not interfere with the sedimentation of nanowires. After 4 hours, cells were fixed, stained for cell F-actin and DNA, and imaged using fluorescence microscopy, and brightfield microscopy.

### Statistical analysis

NW exposure effects were evaluated for each NW group using one-way analysis of variance (ANOVA) followed by pairwise mean comparison using *Tukey* test. The statistical analysis was performed with OriginPro2017 (OriginLab, Northampton, MA). Statistical significance was considered at p<0.05, p<0.01, and p<0.001.

## Results and discussion

### Nanowire exposure

**Fig 1:**
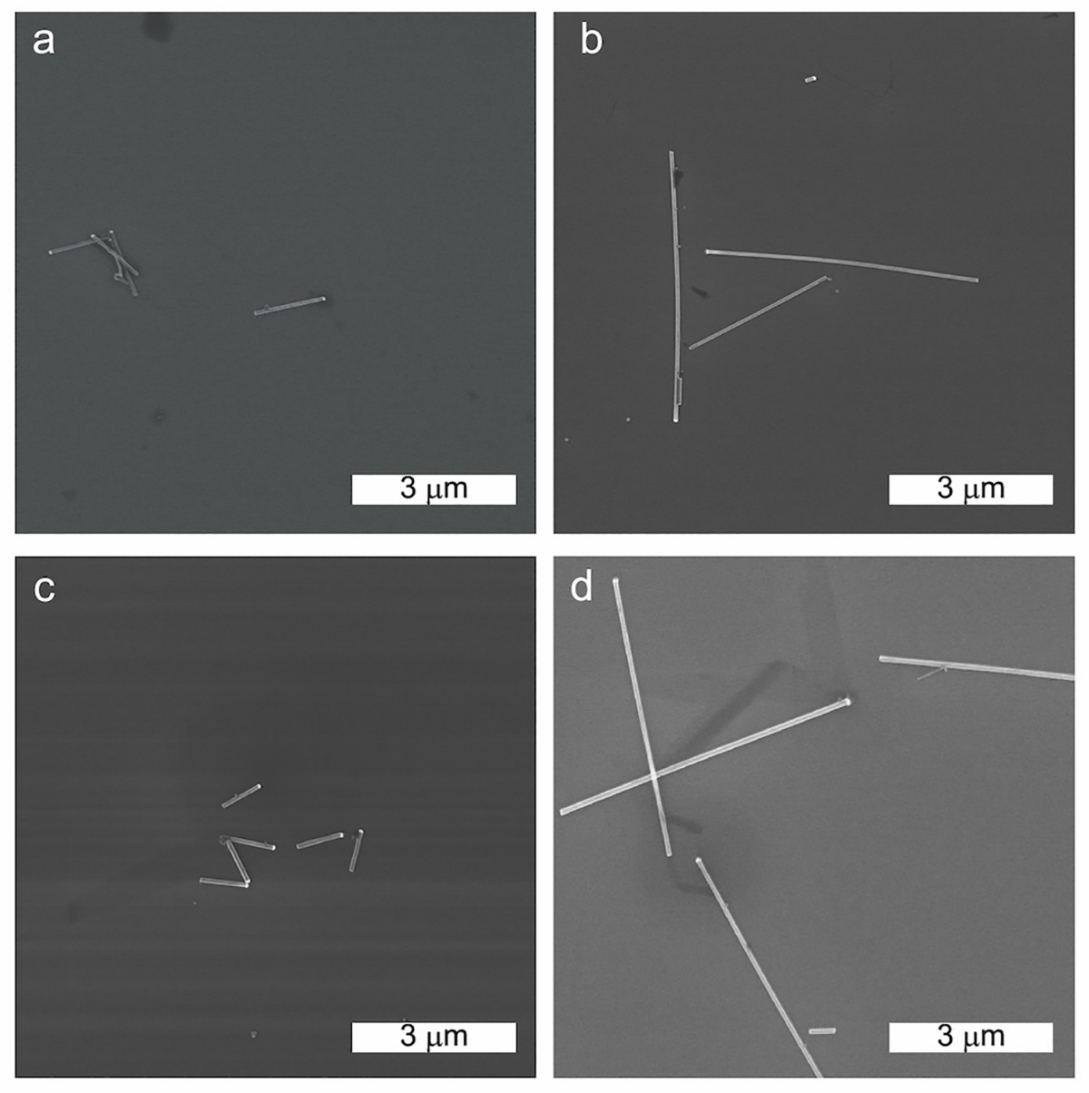
Scanning electron microscope images of the nanowires tested in this study. a) 60 nm diameter, 1 μm long nanowires; b) 60 nm diameter, 5 μm long nanowires; c) 80 nm diameter, 1 μm long nanowires and d) 80 nm diameter, 5 μm long nanowires.

Four groups of nanowires of different nominal diameters d and length L were used in this study: d = 60 nm and L = 1 μm; d = 60 nm and L = 5 μm; d = 80 nm and L = 1 μm; d = 80 nm and L = 5 μm. The diameter and length distribution of each of the 4 nanowire populations were investigated using SEM after sonication (S2 Fig). Fig 1 shows representative SEM images of the nanowires after sonication. A549 cells were exposed to the different nanowire groups at a final nanowire surface concentration of 6.9× 10^−7^ m^2^/mL. Nanowires were coated with a 5 nm Al_2_O_3_ layer to mimic nanowires that are integrated in a optoelectronic device.

After 48 h cells were fixed and stained for DNA and actin, and imaged using fluorescence microscopy. Nanowires were visualized using brightfield microscopy (see S3 Fig). In what follows, the given time points correspond to the elapsed time after the beginning of the nanowire exposure.

### Cell proliferation and nuclear morphology

**Fig 2:**
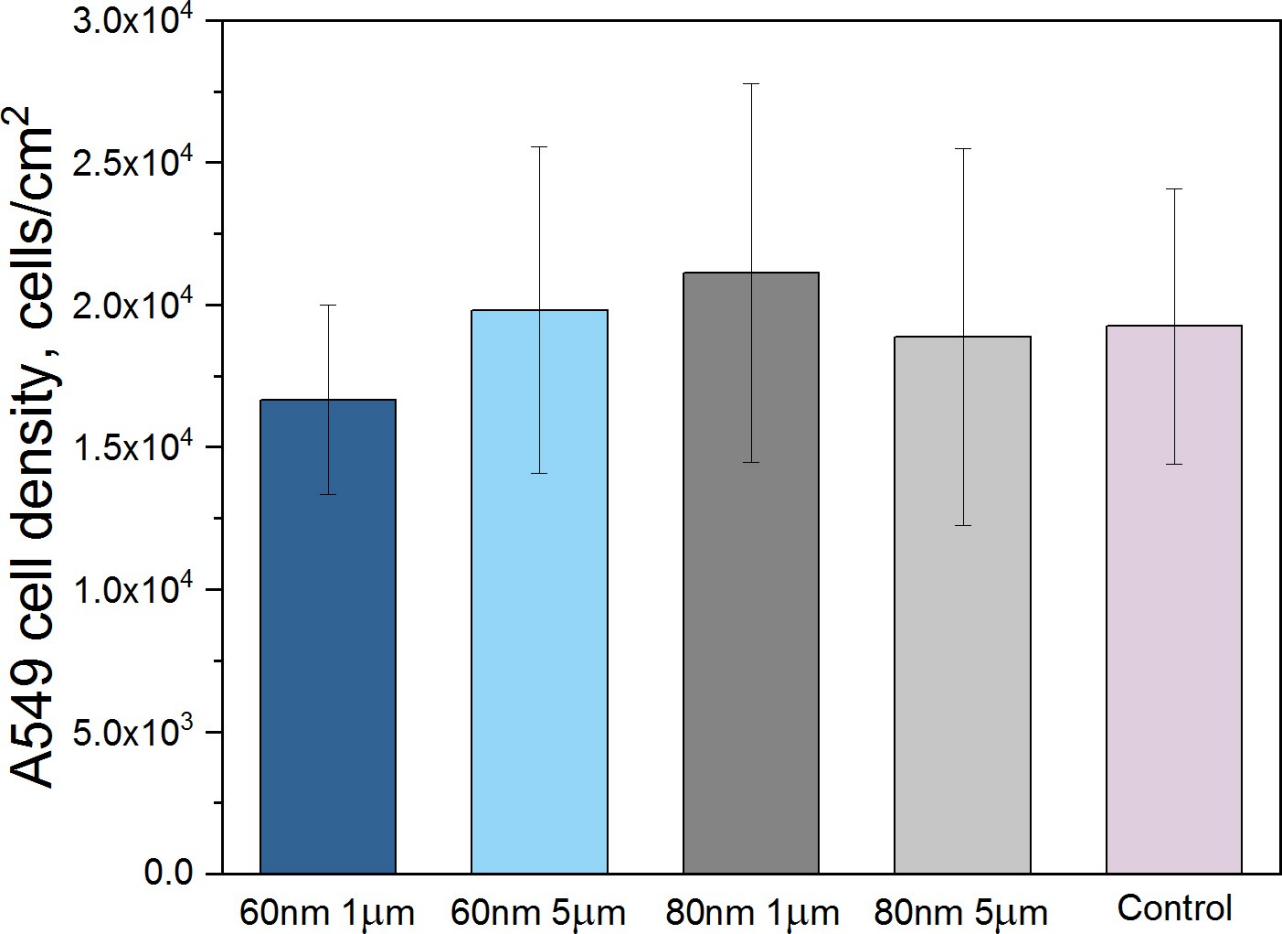
A549 cell density. Density of cells exposed to nanowires and controls, assessed 48h after the beginning of the exposure. No significant difference in cell density between the groups could be found (According to one-way ANOVA statistical analysis p<0.05).

The cell proliferation rate was assessed by quantifying the density of cells exposed to the 4 nanowire groups 48 h after the beginning of the nanowire exposure, and comparing it that of cells not exposed to nanowires (Fig 2). The cell density is similar for all groups, including the controls. This shows that the presence of nanowires does not influence the rate of cell proliferation.

The cell nuclear morphology and number of nuclei was assessed using fluorescence microscopy. No significant differences could be found between exposure and control groups, except in number of nuclei for 5 μm long nanowires of both diameters, which constituted a small fraction of all cells observed (< 10%, S4 Fig).

### Nanowire distribution

**Fig 3:**
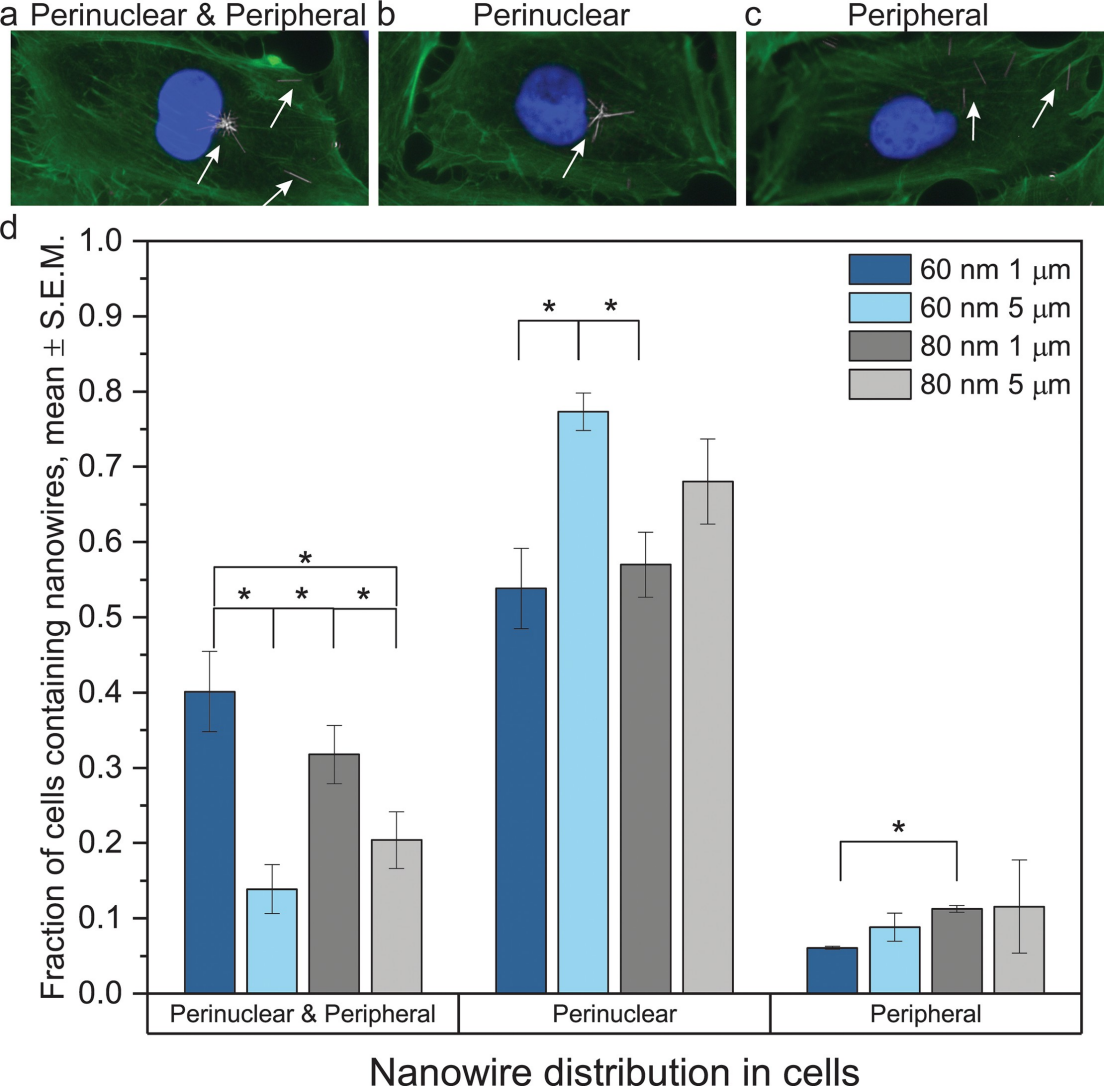
Nanowire distribution within cells. a-c) Fluorescence microscopy images of cells containing nanowire in different regions. d) Nanowire distribution in cells 48 h after the beginning of the nanowire exposure, for different nanowire geometries. (*: p<0.05, ***: p<0.001, one way ANOVA). Most cells containing nanowires have nanowires located in the perinuclear region.

We have verified that the nanowires were indeed internalized using confocal microscopy (S5 Fig).The location of nanowires within cells was assessed at 48 h. Most of the cells containing nanowires have nanowires in the perinuclear region (Fig 3).

Our data is consistent with the results of a previous study on silicon nanowire cellular uptake, showing an active transport of the nanowires to the perinuclear region [45].

**Fig 4:**
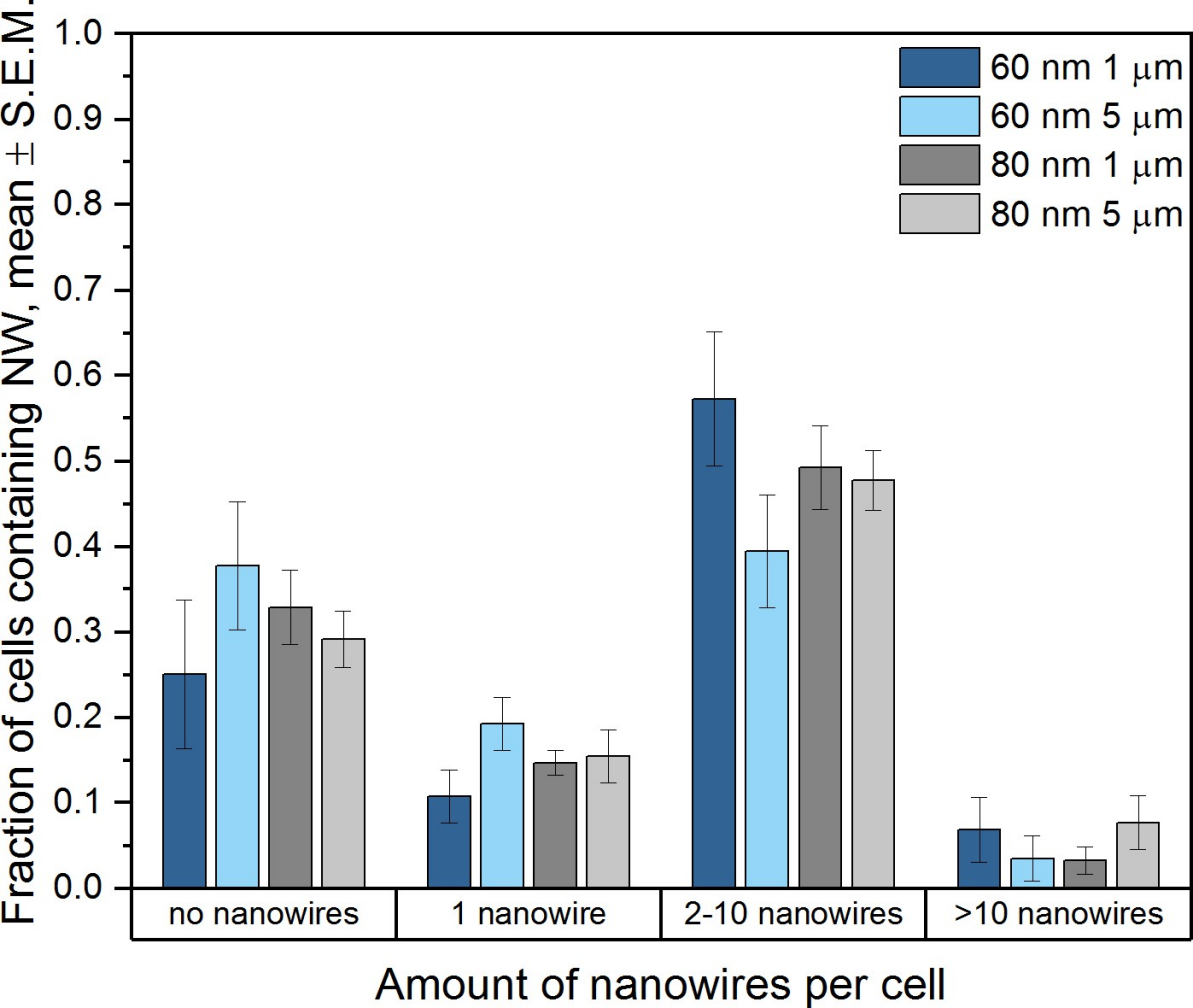
Nanowire quantity per cell. Amount of nanowires found inside cells exposed to nanowires, assessed 48 h after the beginning of the exposure.

The amount of nanowires found in cells was plotted at 48 h (Fig 4). For all groups, the majority of cells contains between 2 and 10 nanowires. No significant differences in nanowire quantity could be found between cells exposed to the different groups of nanowires.

Since we could not find any substantial differences between cells exposed to the 4 different nanowire geometries, in the following evaluation, we have exposed cells to 80 nm diameter, 5 μm long nanowires only.

### Cell viability and ROS levels

**Fig 5:**
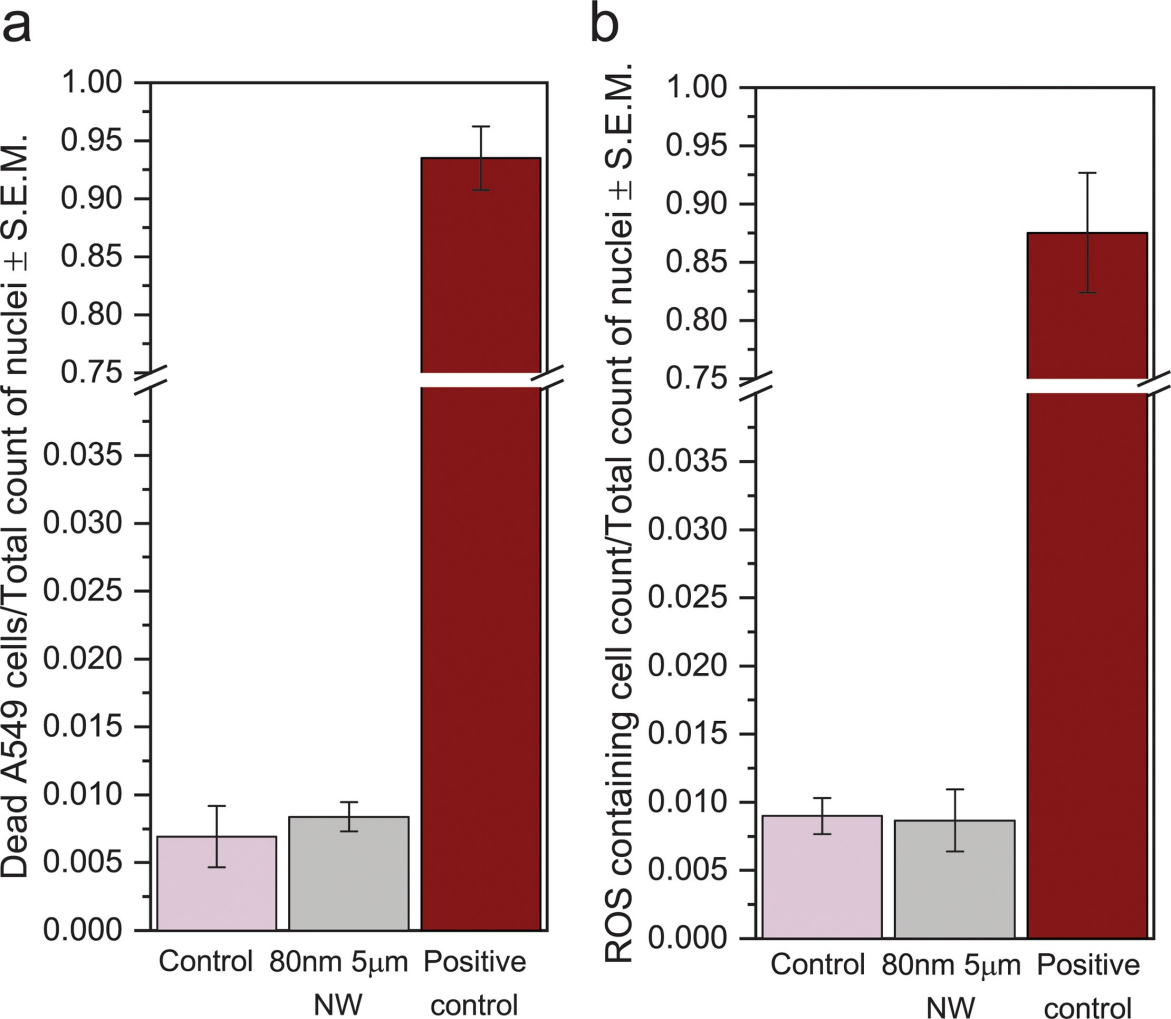
Proportion of dead cells and proportion of ROS-containing cells. **a)** Proportion of dead cells in nanowire-exposed cells and controls, assessed using fluorescein diacetate/propidium iodine live/dead cell assay. b) Proportion of ROS-containing cells. According to one-way ANOVA statistical analysis, differences between exposure and negative control groups were not statistically significant at p<0.05.

We performed a live/dead assay and an intracellular reactive oxygen species (ROS) assay on cells 48 h after the beginning of the nanowire exposure (Fig 5). We have verified that the fluorescence signal is not affected by any interactions of the nanowires with the chemicals used in the assays (S6 Fig). The results show that exposing cells to nanowire does not affect their viability, nor their ROS content. Our results are in agreement with the results of a previous study investigating the influence of iron nanowires on HeLa cells [46]. In that study, little effect on the cell viability was observed when cells were cultured in cell medium containing nanowires. It is important to note, however, that this does not imply that lung cells exposed to nanowires *in vivo* would not be affected. Indeed, the present *in vitro* experiments cannot mimic the immune system response to nanowires, which is a major factor in fiber pathogenicity [47].

We have also investigated the cell motility using phase holographic microscopy [48] and our results show that it is not affected by exposure to nanowires (S7 Fig).

### Nanowire internalization pathways

**Fig 6:**
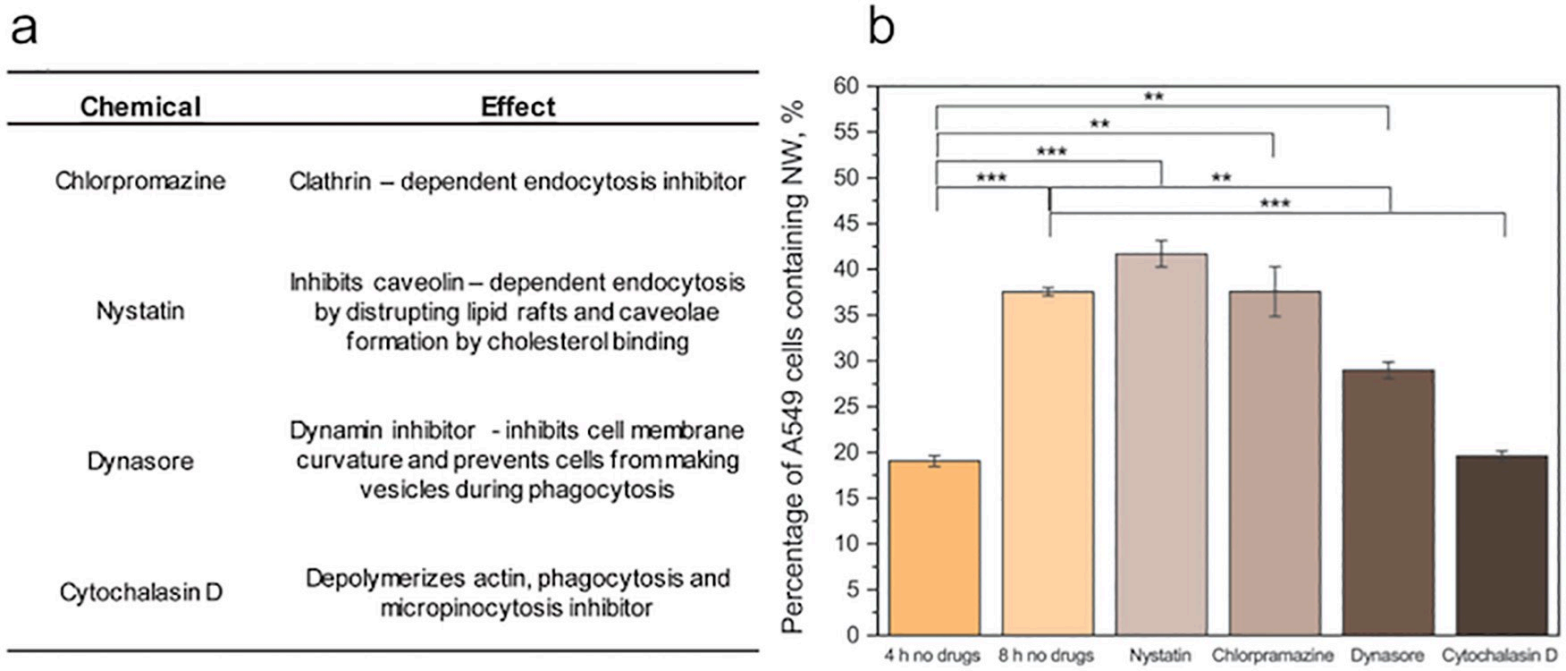
Nanowire uptake mechanisms. a) Effects of the drugs used in this study to block specific pathways. b) Effects of the different drugs on nanowire uptake 8 h after the beginning of nanowire exposure corresponding to 4 h after the beginning of the drug application. The addition of Dynasore and Cytochalasin D leads to a decrease in the percentage of cells containing nanowires, suggesting that the internalization takes place via phagocytosis and macropinocytosis. (**: p<0.01, ***: p<0.001, one way ANOVA).

In order to investigate the mechanisms of nanowire internalization, we have blocked specific internalization pathways 4 hours after the beginning of the exposure and quantified the internalization at 8 h. The choice of 4–8 hour interval was motivated by the fact that this corresponds approximately to the time needed for nanowires to sediment down 1 mm, which is the height of cell medium in the petri dish [49]. This choice was supported by the fact that the 4–8 hour interval corresponds to the regions where internalization as a function of time is the steepest, i.e. where the internalization rate is at its peak (S8 Fig). The main possible internalization pathways for nanowires are phagocytosis, macropinocytosis, clathrin-dependent endocytosis and caveolin-dependent endocytosis. Different compounds were used to block one or several pathways (Fig 6A). Incubating cells with dynasore, which prevents membrane curvature through dynamine inhibition, resulted in a reduced nanowire uptake (Fig 6B). Incubating the cells with cytochalasin D, which prevents actin polymerization, resulted in an even more pronounced reduction in nanowire uptake (Fig 6B). In contrast, incubating cells in nystatin and chlorpromazine, inhibitors of caveolin-dependent endocytosis and clathrin-dependent endocytosis, respectively, did not have any effect on nanowire internalization (Fig 6B). These results suggest that the nanowire uptake is taking place through both phagocytosis and macropinocytosis, which is in agreements with previous studies of silicon and magnetic nanowires [45,50].

Cargos internalized through phagocytosis end up in lysosomes for destruction. In contrast, when engulfed through macropinocytosis, the cargo is first located in macropinosomes, which, after maturation, either fuse back with the cell membrane or fuse with lysosomes. In order to assess whether nanowires are surrounded by cellular compartments, we have stained cells for EEA-1 (labeling macropinosomes and early endosomes [51]) at 8 hours, and LAMP-1 (labeling lysosomes[50,52]) at 8 h and 48 h. In all the images we have acquired, we did not see any nanowire colocalizing with these markers (S9 Fig).

Our results contrast with the ones of previous studies were some nanowires were found surrounded by lysosomes[45,50]. One of these studies suggests that nanowires are released from the lysosomes, although this has not been directly observed [45]. We have previously investigated the effects of injecting nanowires in the rat brain, and found that the nanowires are located inside macrophages [28,53]. In those studies, lysosomes were not stained and we could not determine the fate of the nanowires in terms of cellular compartments. However, GaP nanowires were found to be degraded within the macrophages overtime, suggesting that, in that case, they were located inside lysosomes. Another study on the internalization of iron nanowires by HeLa cells shows that short (<500 nm) nanowire are located inside cellular compartment, whereas longer nanowires (μ1 μμm) are located in the cytosol [46], which is similar to what we have observed for the 5 μm long nanowires. In that study, spontaneous membrane perforation by long nanowires was proposed to explain the difference between the distribution of short and long nanowires in the cells.

To explore whether spontaneous membrane perforation could take place, we can estimate and compare the force *F*_*NW*_ exerted by a GaP nanowire on the cell membrane when sedimenting on a cell:

$$F_{NW}=(\rho_{GaP}-\rho_{medium})\;V_{NW}\;g∼8\times 10^{-16}N$$

where *ρ_GaP_* is the density of GaP (4.14 g/mL), *ρ_medium_* is the density of the culture medium (1 g/mL), V_NW_ is the volume of a nanowire (2.53×10^−20^ m^3^ for a 80 nm diameter, 5 μm long nanowire), and g is the gravitational acceleration (9.8 m×s^-2^). We can compare this to the force *F*_*cell*_ exerted by one nanowire on the cell membrane when a cell sediments on a vertical nanowire array:

$$F_{cell}=\frac{(\rho_{cell}-\rho_{medium})\;V_{cell}\;g}{N_{NW}}∼3\times 10^{-12}N$$

where *ρ_cell_* is the cell density (1.05 g/mL), *V_cell_* is the volume of a cell (≈100 μm^3^), *N_NW_* is the number of nanowires under a cell (100 for a typical 1 μm^-2^ nanowire density array, assuming a 100 μm^2^ cell area).

The force exerted on the cell membrane by a sedimenting nanowire is negligible compared to the force exerted by a vertical nanowire on the cell membrane during cell sedimentation on a vertical nanowire array. Since, in the latter case, the literature is unanimous, reporting that the membrane of cells cultured on vertical nanowire arrays is rarely pierced [8,54,55], we can assume that, in the present study, the membrane remains intact when nanowires sediment onto cells and that nanowires therefore do not enter cells by direct membrane perforation. This assumption is also reaffirmed when comparing the pressure *P*_*NW*_, exerted by a nanowire of radius *r* = 40 nm, sedimenting on a cell, to the pressure found in the literature, required for a cylindrical atomic force microscope tip to pierce the membrane (1 nN for a tip of 200 nm diameter, translating to 3.18 10^4^ Pa [56]). $P_{Nw}=\frac{F_{Nw}}{{\pi r}^{2}}=1.610^{-1}\;Pa$, i.e. *P*_*NW*_ is five orders of magnitude lower than the pressure necessary to pierce the membrane. Therefore, spontaneous membrane perforation can be ruled out as internalization mechanism of nanowires. Instead, an active mechanism must be considered.

In the present study, the fact that we did not find any nanowire associated with cellular compartments suggests a deviation from normal phagocytosis/macropinocytosis at an early stage. Such an early endosomal escape, combined with the fact that we did not observe any effects of nanowire internalization on cell viability and ROS level, could make free floating nanowires a promising tool for transfection [57]. However, more studies would be required to verify a possible early endosomal escape for nanowires.

## Conclusions

In summary, we have suspended nanowires in the cell medium of cultured A549 cells and investigated the fate of the nanowires and their effects on cell properties. Our results show that nanowires are engulfed by the cells and end up, for the most part, in the cell perinuclear region. The intracellular presence of nanowires does not affect the cell viability, proliferation and motility. The nanowires are internalized through processes requiring dynamin and actin polymerization, suggesting that phagocytosis and macropinocytosis are involved. In our study, nanowires were not found surrounded by macropinosomes or lysosomes, however, this needs to be further confirmed by additional studies. An early nanowire escape from cellular compartments would open for the possibility to use nanowires for cellular transfection, for which early endosome escape is a prerequisite to a successful transfection.

## Supporting information

S1 Fig Raman spectrum of a GaP substrate coated with Al_2_O_3_. The spectrum shows a GaP scattering peak at 405 cm^-1^ from longitudinal optical (GaP_LO_) as well as at 370 cm^-1^ from transverse optical (GaP_TO_) phonons. The spectrum also shows a Al_2_O_3_ crystal peak at 750 cm^-1^. Raman spectrum was acquired using 725 nm laser for 20 seconds. The spectrum is normalized for 1 s. Analysis was performed by fitting the spectrum with Lorentzian functions using the Origin software.
(TIFF)

S2 Fig Diameter (top panels) and length (bottom panels) distributions of the nanowires tested in this study.
(TIF)

S3 Fig Representative images of A549 cells 48 hours after exposure to nanowires. Cell F-actin and nuclei were imaged using fluorescence microscopy, nanowires were imaged using brightfield microscopy (note that the bright nanowires on dark background images are brightfield microscopy images that were inverted using ImageJ).
(TIFF)

S4 Fig Multinuclear cells and nucleus morphology. Number of nuclei (a) and nucleus morphology (b) for cells exposed to nanowires and controls, assessed 48 h after the beginning of the exposure. (*: p<0.05, **: p<0.01, one way ANOVA).
(TIF)

S5 Fig Nanowire internalization. Confocal microscopy scans of fixed A549 cells fluorescently labelled for F-actin (in red, via Phalloidin-STAR635P), the cell nucleus (in green, via Hoechst 33342), and incubated with Al_2_O_3_ GaP nanowires (in blue, reflected signal) for 48h. The uptake of NWs by the cells is clearly visible. Please note the rectangular pixel size of (50 x 250) nm^2^ in the axial (XZ) scans. Raw image data with color channel brightness levels adjusted for visibility are shown. Scale bars: 10 **μ**m.
(TIFF)

S6 Fig Lack of interactions of the nanowires with the chemicals used in the live/dead assay. Nanowires without cells were incubated with the chemicals from live/dead assay and the nanowires were imaged using the same setting as when performing the live/dead assay. The dark images in the FDA and PI detection channels show that the chemicals do not interact with the nanowires.
(TIFF)

S7 Fig Motility of cells exposed to nanowires and control cells, assessed using phase holographic microscopy. (According to one-way ANOVA statistical analysis, differences between exposure and control groups were not statistically significant at p<0.05).
(TIFF)

S8 Fig Time scale of the nanowire internalization. Proportion of cells with internalized nanowires, as a function of time after the beginning of nanowire exposure.
(TIFF)

S9 Fig Nanowire localization in the cytosol. Representative optical microscopy images of A549 cells stained fluorescently for EEA-1 at 8 hours and LAMP-1 at both 8 and 48 hours (red). The nanowires are visualized through bright field microscopy (central panels, white).
(TIFF)
