# Supplementary figures and images for: Uptake of nanowires by human lung adenocarcinoma cells
Source: PLoS One. 2019 Jun 21;14(6):e0218122. doi: 10.1371/journal.pone.0218122 (PMC6588221; doi:10.1371/journal.pone.0218122)

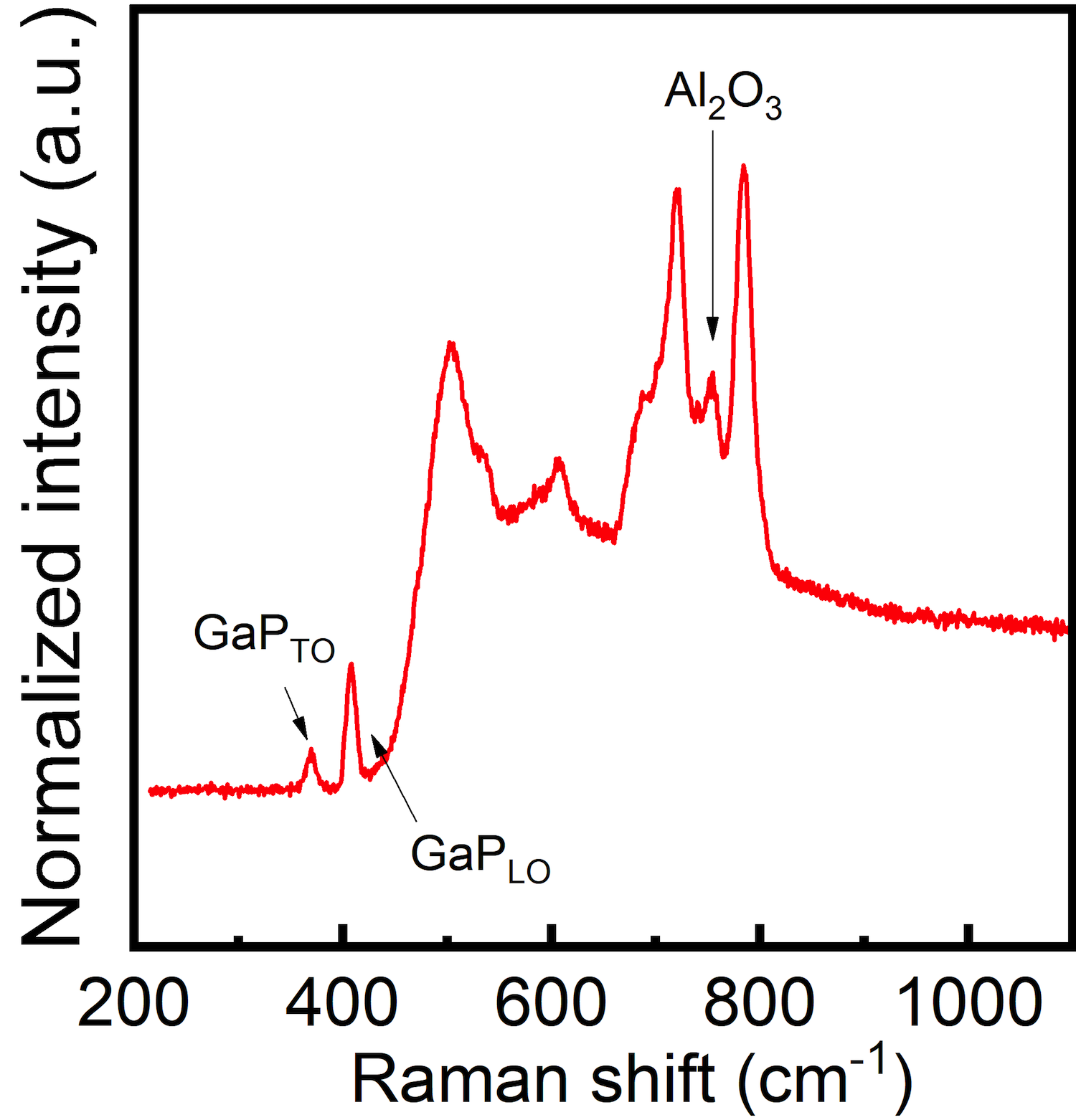

Supplement: S1 Fig — The spectrum shows a GaP scattering peak at 405 cm-1 from longitudinal optical (GaPLO) as well as at 370 cm-1 from transverse optical (GaPTO) phonons. The spectrum also shows a Al2O3 crystal peak at 750 cm-1. Raman spectrum was acquired using 725 nm laser for 20 seconds. The spectrum is normalized for 1 s. Analysis was performed by fitting the spectrum with Lorentzian functions using the Origin software. (TIFF) [file pone.0218122.s001.tiff]

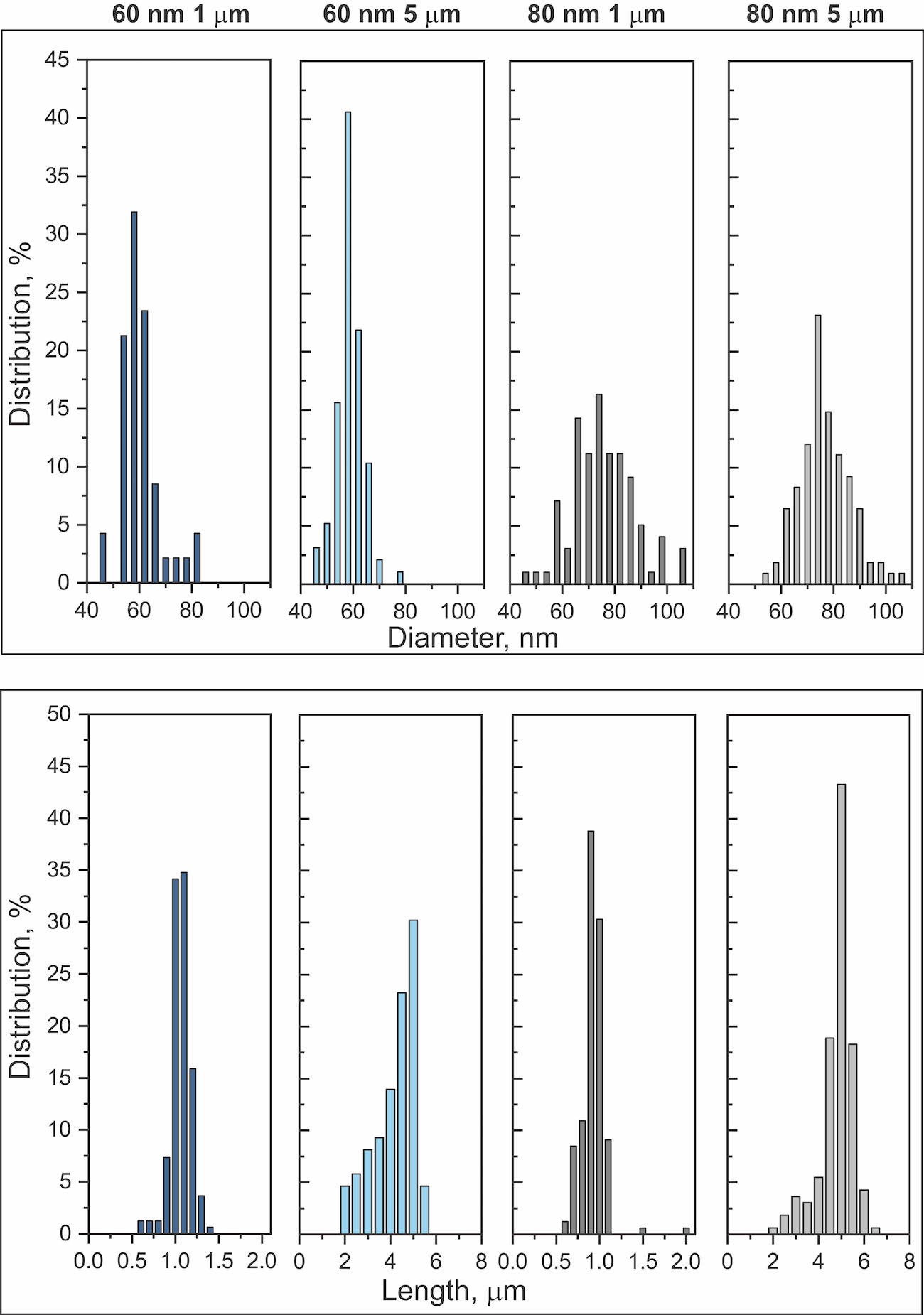

Supplement: S2 Fig — Diameter (top panels) and length (bottom panels) distributions of the nanowires tested in this study. (TIF) [file pone.0218122.s002.tif]

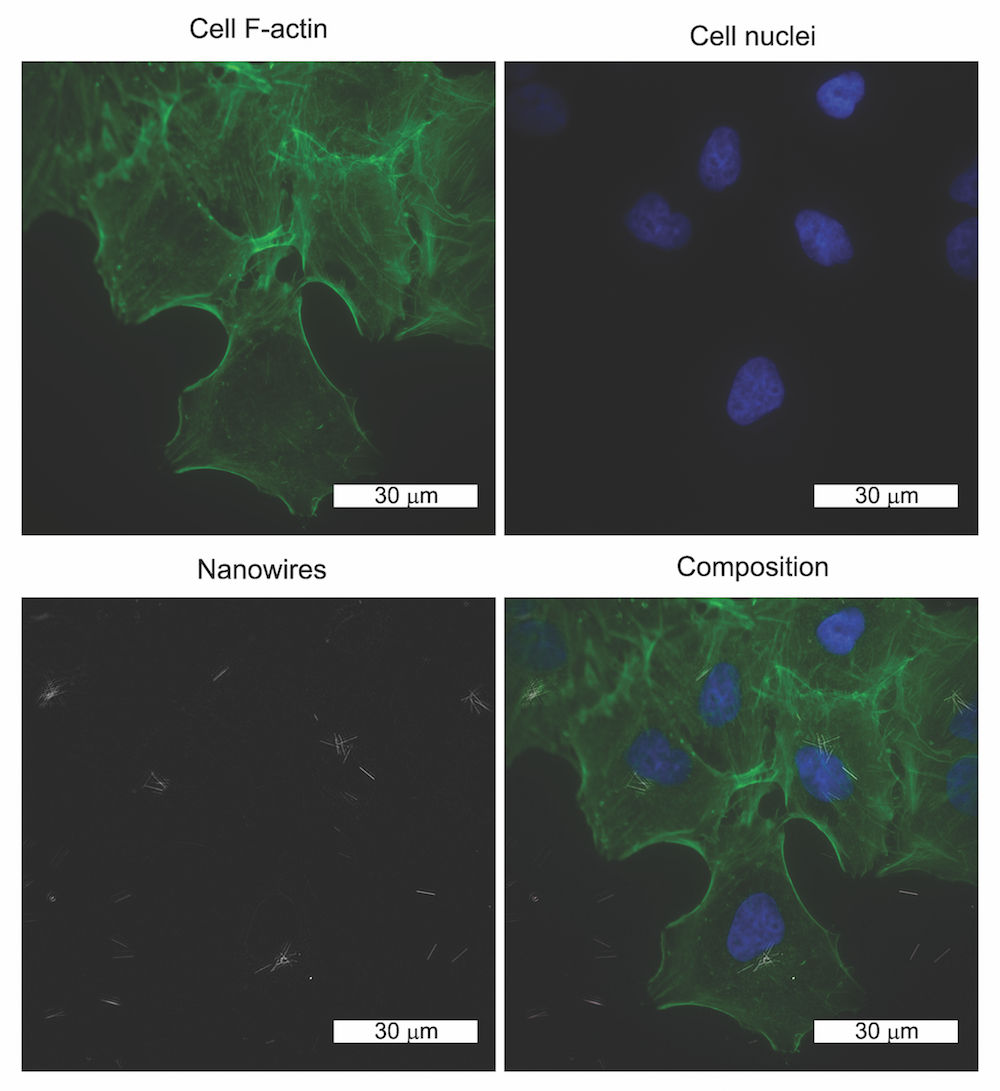

Supplement: S3 Fig — Cell F-actin and nuclei were imaged using fluorescence microscopy, nanowires were imaged using brightfield microscopy (note that the bright nanowires on dark background images are brightfield microscopy images that were inverted using ImageJ). (TIFF) [file pone.0218122.s003.tiff]

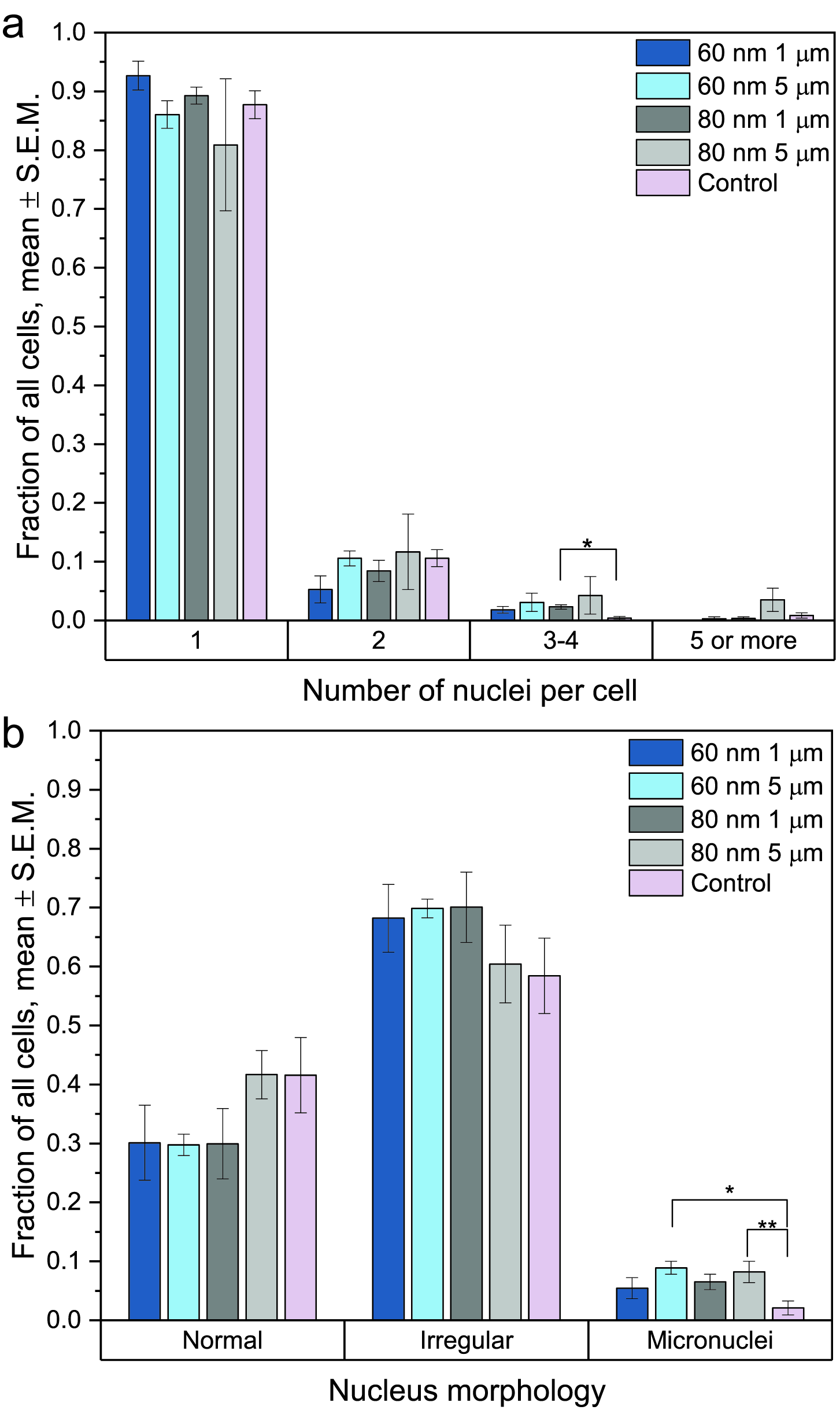

Supplement: S4 Fig — Number of nuclei (a) and nucleus morphology (b) for cells exposed to nanowires and controls, assessed 48 h after the beginning of the exposure. (*: p<0.05, **: p<0.01, one way ANOVA). (TIF) [file pone.0218122.s004.tif]

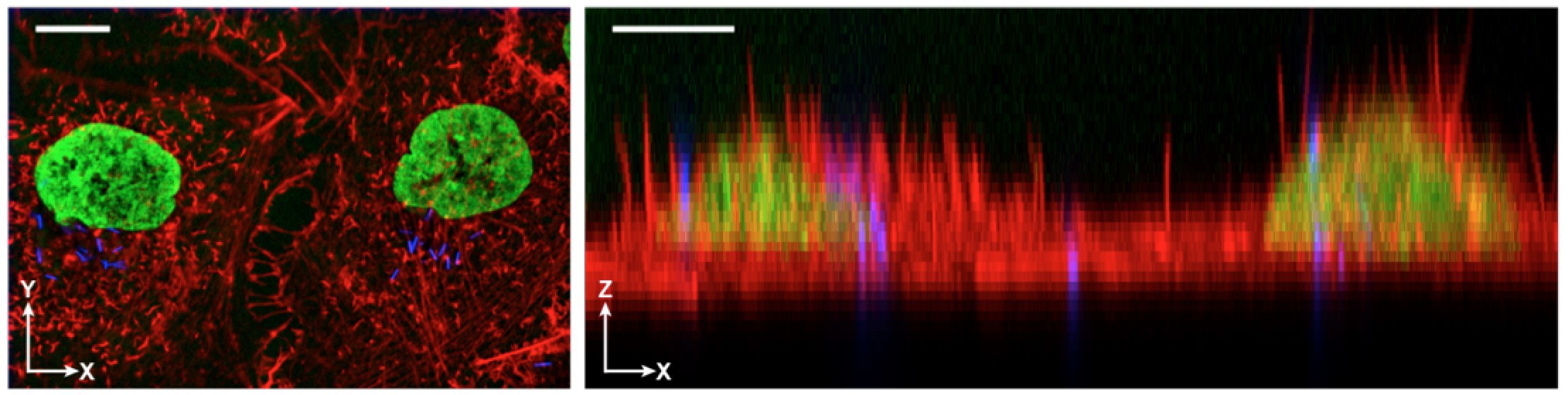

Supplement: S5 Fig — Confocal microscopy scans of fixed A549 cells fluorescently labelled for F-actin (in red, via Phalloidin-STAR635P), the cell nucleus (in green, via Hoechst 33342), and incubated with Al2O3 GaP nanowires (in blue, reflected signal) for 48h. The uptake of NWs by the cells is clearly visible. Please note the rectangular pixel size of (50 x 250) nm2 in the axial (XZ) scans. Raw image data with color channel brightness levels adjusted for visibility are shown. Scale bars: 10 μm. (TIFF) [file pone.0218122.s005.tiff]

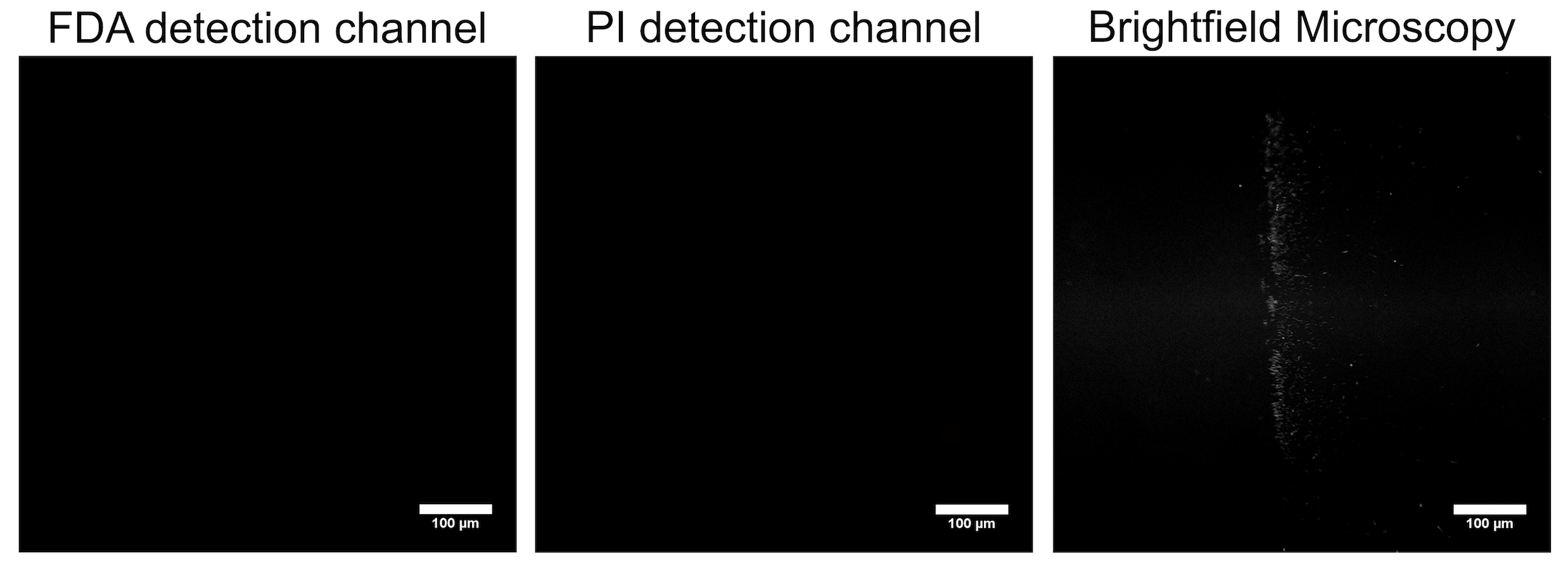

Supplement: S6 Fig — Nanowires without cells were incubated with the chemicals from live/dead assay and the nanowires were imaged using the same setting as when performing the live/dead assay. The dark images in the FDA and PI detection channels show that the chemicals do not interact with the nanowires. (TIFF) [file pone.0218122.s006.tiff]

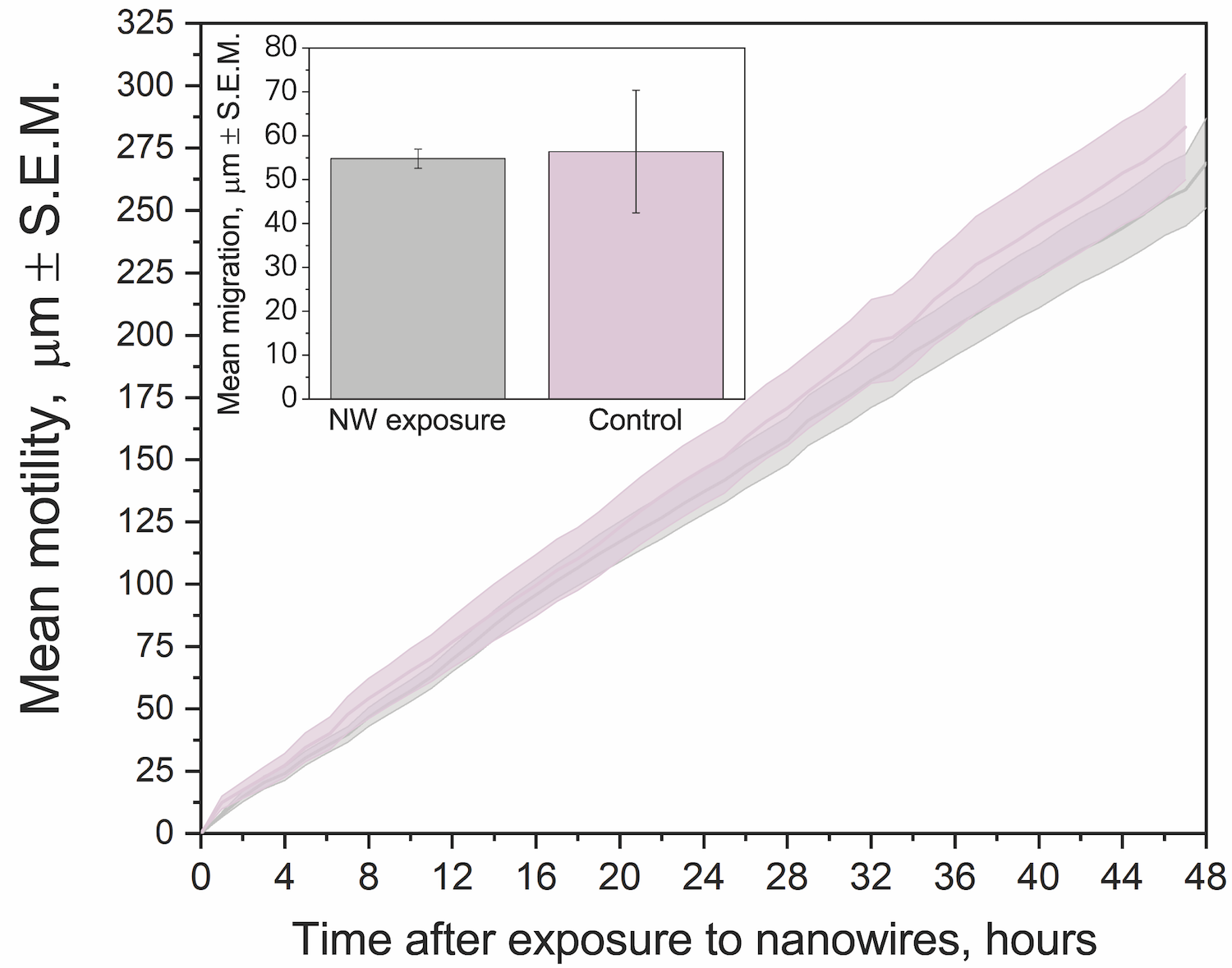

Supplement: S7 Fig — (According to one-way ANOVA statistical analysis, differences between exposure and control groups were not statistically significant at p<0.05). (TIFF) [file pone.0218122.s007.tiff]

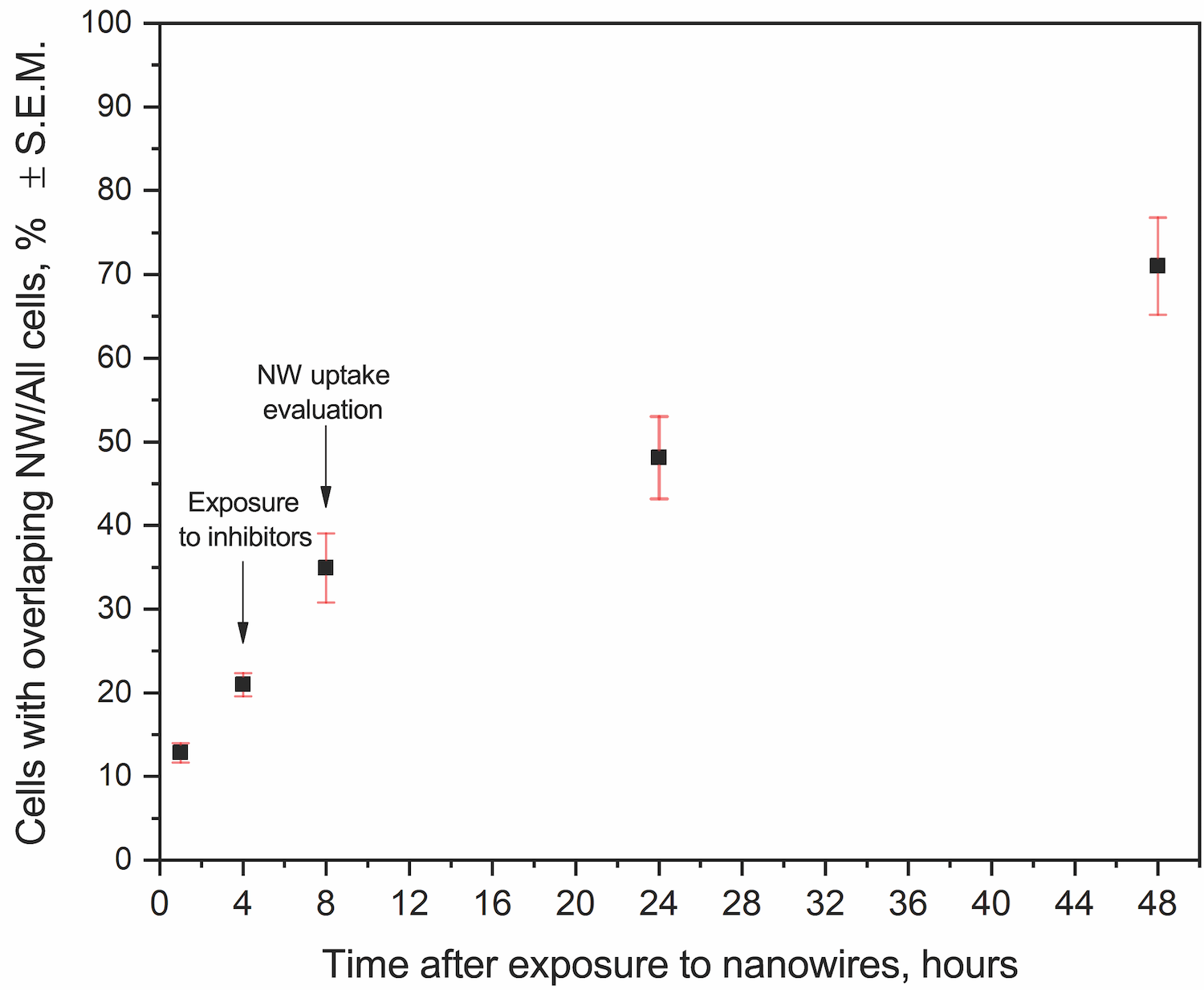

Supplement: S8 Fig — Proportion of cells with internalized nanowires, as a function of time after the beginning of nanowire exposure. (TIFF) [file pone.0218122.s008.tiff]

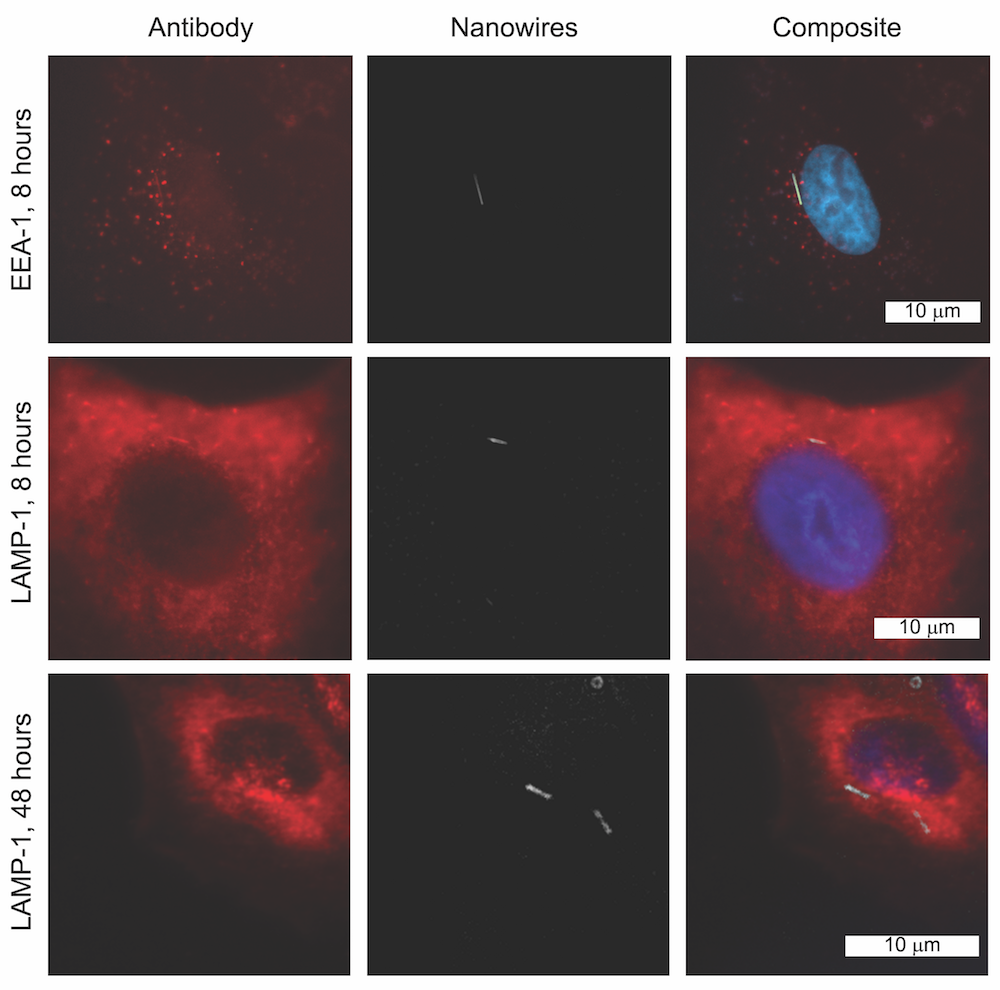

Supplement: S9 Fig — Representative optical microscopy images of A549 cells stained fluorescently for EEA-1 at 8 hours and LAMP-1 at both 8 and 48 hours (red). The nanowires are visualized through bright field microscopy (central panels, white). (TIFF) [file pone.0218122.s009.tiff]
